# The influence of structured reporting on the accuracy of head and neck sonographies

**DOI:** 10.1038/s41598-026-43561-1

**Published:** 2026-03-10

**Authors:** Johannes Matthias Weimer, Julian Künzel, Christoph Raczeck, Mohamed Hodeib, Tim Koppen, Liv Weimer, Anna Levi, Maximilian Rink, Sven Becker, Benjamin Philipp Ernst

**Affiliations:** 1 Rudolf-Frey Teaching Department, University Medical Center of the Johannes Gutenberg University Mainz, Langenbeckstraße 1, 55131 Mainz, Germany; 2 Department of Internal Medicine I, University Medical Center of the Johannes Gutenberg University Mainz, Langenbeckstraße 1, 55131 Mainz, Germany; 3 Department of Otorhinolaryngology, University Hospital Regensburg, Franz-Josef-Strauß-Allee 11, 93053 Regensburg, Germany; 4 Department of Otorhinolaryngology, University Medical Center Bonn, Venusberg-Campus 1, 53127 Bonn, Germany; 5 Department of General, Visceral and Thoracic Surgery, German Armed Forces Central Hospital of Koblenz, Rübenacher Straße 170, 56072 Koblenz, Germany; 6 Department of Radiation Oncology and Radiotherapy, University Medical Center of the Johannes Gutenberg University Mainz, Langenbeckstraße 1, 55131 Mainz, Germany; 7 Department of Otorhinolaryngology, Goethe-University Frankfurt, University Medical Center, Theodor-Stern-Kai 7, 60590 Frankfurt, Germany; 8 Department of Otorhinolaryngology, Head and Neck Surgery, University of Tübingen Medical Center, Elfriede- Aulhorn-Straße 5, 72076 Tübingen, Germany

**Keywords:** Ultrasonography, Report Quality, Structured Reporting, Report Accuracy, Ultrasound Training, Health care, Medical research

## Abstract

The use of structured reports (SR) has been shown to improve report completeness, time efficiency and interrater reliability in head and neck sonography (HNS). However, no data exists to date on the influence on report accuracy in terms of correct interpretation of findings. The aim of this study was to evaluate report accuracy as well as completeness using SR and free text reports (FTR). 128 participants of certified HNS courses were randomly assigned to create either SRs or FTR of predefined clinical cases. Demographic data, clinical training level and ultrasound experience of participants were documented prior to randomization. Each case included ultrasound images and clinical histories representing typical pathologies in HNS. Reports were independently evaluated by DEGUM-certified otorhinolaryngologists using standardized templates with respect to accuracy and completeness. SR demonstrated significantly higher accuracy (*p* < 0.001) as well as completeness (*p* < 0.001) compared to FTR. A strong positive correlation was found between completeness and accuracy using SR (*r* = 0.30, *p* < 0.01) only. Additionally, use of SR was the only significant predictor of improved report accuracy (*p* < 0.01) and completeness (*p* < 0.01). Structured reporting significantly improves both completeness and accuracy in head and neck ultrasound reports. The use of SR may contribute to more consistent reporting quality in clinical and educational settings.

**Supplementary Information:**

The online version contains supplementary material available at 10.1038/s41598-026-43561-1.

## Introduction

Ultrasound imaging is an essential diagnostic tool in numerous medical disciplines. Ongoing technological advancements have broadened its clinical utility, enabling point-of-care confirmation or exclusion of a wide range of suspected diagnoses in a non-invasive, radiation-free, and cost-effective manner. In this context, head and neck sonography (HNS) has emerged as a first-line diagnostic modality for multiparametric evaluating diverse pathologies and assisting with interventional procedures in both otorhinolaryngology and maxillofacial surgery^1,2^.

Despite its many advantages over modalities such as magnetic resonance imaging (MRI) and computed tomography (CT), sonography remains highly operator-dependent^3^. The reliance on examiner expertise is particularly critical in the preoperative assessment of soft tissue pathologies in head and neck surgery, where insufficient ultrasound findings may lead to intraoperative complications, prolonged surgical time, or even necessity of re-operations^4^.

To mitigate such risks, systematic training curricula in ultrasound should not only teach technical skills but also emphasize standardized examination protocols and high-quality reporting. A major challenge in current practice remains the lack of standardization in the structure and content of HNS reports, which leads to considerable variability in report quality^5,6^. Although national and international societies have issued training recommendations, educational standards and reporting practices in HNS still differ significantly across institutions^7–10^.

Non-standardized free text reports (FTR) can result in loss of critical information, potentially causing misinterpretations and dissatisfaction among referring physicians^11^. Structured reporting (SR) has been shown to enhance both report quality and time efficiency across different levels of training and in various clinical contexts—including sonography, surgical planning, and interdisciplinary diagnostic processes^7,11–17^.

Moreover, initial studies have demonstrated that SR can significantly improve inter-rater reliability (IRR) and inter-rater agreement (IRA), thereby reducing one of its most criticized disadvantages^18^. While this benefit has been confirmed for CT examination of the neck^19^ studies focusing on other anatomical regions have shown mixed results, depending largely on the examiner’s expertise and the structured nature of their reporting approach^20^. In addition to IRR and IRA, the concept of accuracy in diagnostic imaging is gaining increasing relevance^21,22^. In measurement theory, precision describes the consistency or reproducibility of results obtained under standardized conditions and is conceptually distinct from accuracy, which refers to correctness with respect to true clinical outcomes^23^. Accordingly, the present study introduces report accuracy as a distinct construct, defined as the correctness of reported items and terminology compared with an expert-derived master report.

While previous studies on structured reporting in head and neck ultrasound primarily focused on report completeness, time efficiency, and inter-rater reliability or agreement, the impact of structured reporting on the accuracy of reported content has not been systematically investigated.

The present study addresses this gap by introducing an item-based, reference-standard–driven assessment of report accuracy, defined as the correctness and internal consistency of reported items and terminology relative to an expert-derived master report, in a randomized educational setting. By jointly evaluating completeness and accuracy and analyzing their interrelationship, this study extends existing evidence on structured reporting beyond structural quality metrics toward a more granular assessment of reporting content.

## Methods

### Study design

Ethics approval was obtained by the Institutional Review Board. This prospective randomized study was conducted from 2023 to 2024. A total of 128 participants of an accredited course on head and neck ultrasound by the German Society for Ultrasound in Medicine (DEGUM) were included. The primary endpoints were defined as report completeness and report accuracy. Inclusion criteria comprised attendance of a DEGUM-certified head and neck ultrasound course, written informed consent, and completion of at least one assigned case. Participants with missing consent or incomplete report submission were excluded.

Participants consisted of residents of various specialties and were accustomed to creating FTRs in their daily clinical routine prior to inclusion in the study, participants’ baseline and individual levels of experience in ultrasound diagnostics were evaluated through a digital evaluation form.

**Fig. 1:**
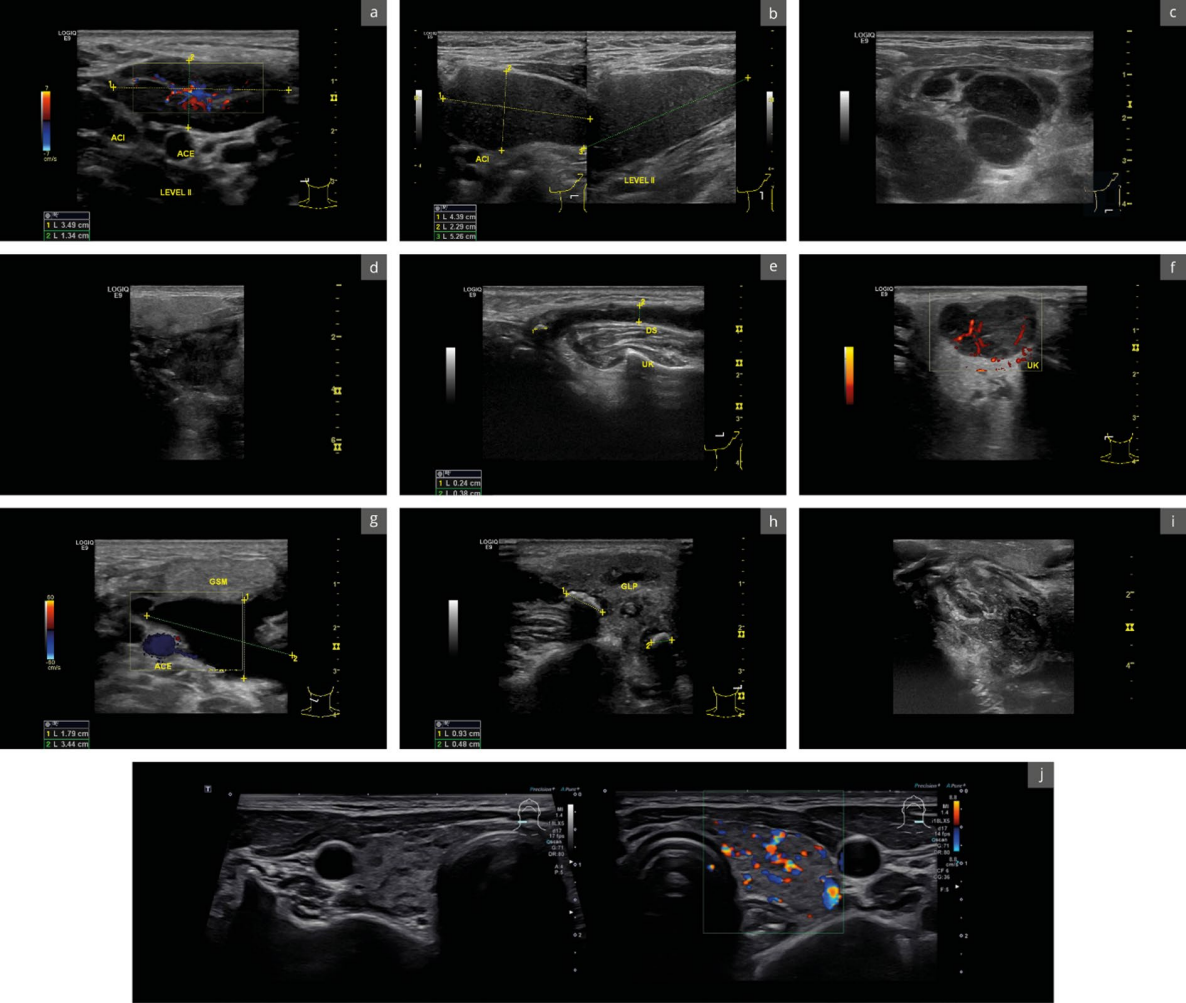
Pathologies to be reported in this study. (**a**) Acute cervical lymphadenitis, (**b**) Branchial cleft cyst, (**c**) Non-Hodgkin lymphoma of the neck, (**d**) Tongue base tumor, (**e**) Sialolithiasis with duct obstruction, (**f**) Pleomorphic adenoma of the parotid gland, (**g**) Plunging ranula, (**h**) Multifragmentary sialolithiasis, (**i**) Peritonsillar abscess, (**j**) Hashimoto’s thyroiditis.

Participants were randomly assigned to two groups to create either FTRs or SRs for this study and received introductory training in the randomized modality. In the following step, each participant received two randomly assigned cases, including patient histories and matching ultrasound images of the respective pathologies. These cases were created by board-certified otorhinolaryngologists with DEGUM Level III certification, based on representative findings from their daily clinical practice as well as on the clinical and surgical results that were acquired along each case. (see Fig. 1 and Supplement 1). All cases were based on adult patients (≥ 18 years of age). Subsequently, participating residents reported upon the allocated cases using FTR or SR as randomized. Structures which were not illustrated within the provided documentation were meant to be reported as unremarkable.

### FTR and SR

In this study, our standard form, which is frequently used for head and neck ultrasound courses was employed to create FTRs. As previously described, an online-based platform (Smart Reporting GmbH, Munich, Germany, https://smart-reporting.com) was used to create a specialized structured reporting template for head and neck ultrasound studies The SR template was created based on the most recent recommendations of the DEGUM. Consequently, it may be used universally for virtually any pathology^7,11–13,24^.

### Report evaluation

For all ten cases, expert-derived master reports were created by board-certified otorhinolaryngologists with DEGUM level III certification. These master reports represented the reference standard for both completeness and accuracy scoring. Each master report included all clinically relevant positive findings as well as explicitly documented negative findings for anatomical regions without pathology, reflecting routine clinical reporting practice.

Subsequently, case-specific evaluation templates were derived directly from the master reports. For completeness assessment, the evaluation templates listed all anatomical regions and report elements that were expected to be mentioned in a standard HNS report, independent of report accuracy. In this scenario, the same scoring system was used for all 10 cases (e.g. all cases utilize same elements and a maximum point score of 37). For accuracy assessment, each anatomical region was subdivided into discrete, objectively assessable items corresponding to the content of the master report. These items included, depending on the region, the correct identification of normal anatomy, exclusion of pathology, and detailed characterization of pathological findings (e.g., number of lesions, size, localization, echogenicity, border definition, and suspected diagnosis). In this scenario, individual scoring systems were developed for each case to reflect the complexity of the pathology at hand. This results in varying maximum point scores depending on the case (e.g. maximum point score of 30–37). To enhance comparability, relative scoring compared to the maximum point score were used rather than the raw absolute point score.

Each evaluable item was assigned a predefined point value reflecting its diagnostic relevance within the respective anatomical context. This weighting approach ensured transparent and reproducible scoring while avoiding subjective global quality ratings. The same structural design principles and scoring logic were applied consistently across all cases, ensuring comparability between reports and between reporting formats. (see Supplement 2). The resulting point values were then set in relation to the maximum achievable score to quantify the degree of completeness and accuracy of the findings.

Subsequently, all reports (*n* = 118 FTRs and *n* = 138 SRs) were anonymized and evaluated by two board- and DEGUM-certified otorhinolaryngologists independently in terms of their individual completeness and accuracy. As mentioned above, scores were compared to the master report regarding whether certain aspects were mentioned (completeness) and whether they were described correctly (accuracy).

To ensure the comparability of findings and to investigate the effects of SR vs. FTR, only cases were included for which both report formats were available in sufficient number, created by different users with documented experience in HNS.

### Statistical analysis and sample size calculation

The sample size of reports needed was calculated based on the anticipated effect size when comparing the proportion of each report type with a accuracy of 80% or higher^25^. Taking prior publications into account, we assumed that 50% of FTRs and 70% of STs would meet these criteria^7,11^. Using a power of 80%, the significance level was set with an α = 0.05. Using these parameters, the minimum number of reports required within this trial was computed to be *n* = 186 (93 reports of each type).

All written forms were digitalized, then analyzed with the evaluation mask and the scores transferred to Microsoft Excel (Microsoft Corp., Redmond, WA, USA). The results of the digital evaluation were also issued to Microsoft Exel. All statistical analyses were performed in Prism 10 (GraphPad Software Inc., Boston, MA, USA). Binary and categorical baseline characteristics are presented as counts and percentages. Continuous variables are reported as means with standard deviations (SD) Categorical variables were analyzed using the chi-squared test. Normality of data distributions was assessed using the Shapiro-Wilk test, ensuring appropriate use of parametric tests. Levene’s test was applied to check for homogeneity of variance. For continuous variables, normally distributed data were analyzed using the unpaired t-test when comparing report completeness and accuracy between groups. Before performing inferential analyses, we examined pairwise correlations between the variables ‘Completeness’ and ‘Accuracy’ and visualized both the effect sizes and their statistical significance. Multivariate linear regression models were constructed to compare the influence of individual factors on the results score of completeness and accuracy. P-values < 0.05 were considered statistically significant.

## Results

### Baseline characteristics

A total of 128 participants were included in the study and randomly assigned to either the FTR (*n* = 58) or SR (*n* = 70) group. Baseline characteristics (see Table 1) did not differ significantly between groups. The mean age was comparable (*p* = 0.78) and the gender distribution was balanced (FTR: 58% female; SR: 61%; *p* = 0.50). Most participants were residents (FTR: 95%; SR: 92%), with only a small proportion being specialists or consultants (*p* = 0.20). The majority had no current DEGUM certification level (FTR: 85%; SR: 88%; *p* = 0.74). Clinical experience, measured by the number of independently performed ultrasound examinations, was similar between groups (FTR: 101 ± 132; SR: 107 ± 106; *p* = 0.76). Participants were primarily otorhinolaryngologists (FTR: 91%; SR: 79%), with a significantly higher proportion of maxillofacial surgeons in the SR group (FTR: 9%; SR: 21%; *p* = 0.04).

**Table 1:** Baseline characteristics of participating residents.

Characteristics	FTR	SR	*p* - value
Age (Mean ± SD)	29.6 ± 4.7	29.8 ± 4.1	0.78
Gender			0.5
Male	0.42	0.39	
Female	0.58	0.61	
Level of clinical training			0.2
Resident	0.95	0.92	
Specialist	0.03	0.06	
Consultant	0.02	0.02	
Specialization			0.04
Otorhinolaryngology	0.91	0.79	
Maxillofacial Surgery	0.09	0.21	
Current DEGUM level (%)			0.74
None	0.85	0.88	
I	0.15	0.12	
II	0	0	
III	0	0	
Independently performed ultrasound examinations (MW ± SD)	101 ± 132	107 ± 106	0.76

### Report completeness and accuracy

**Fig. 2:**
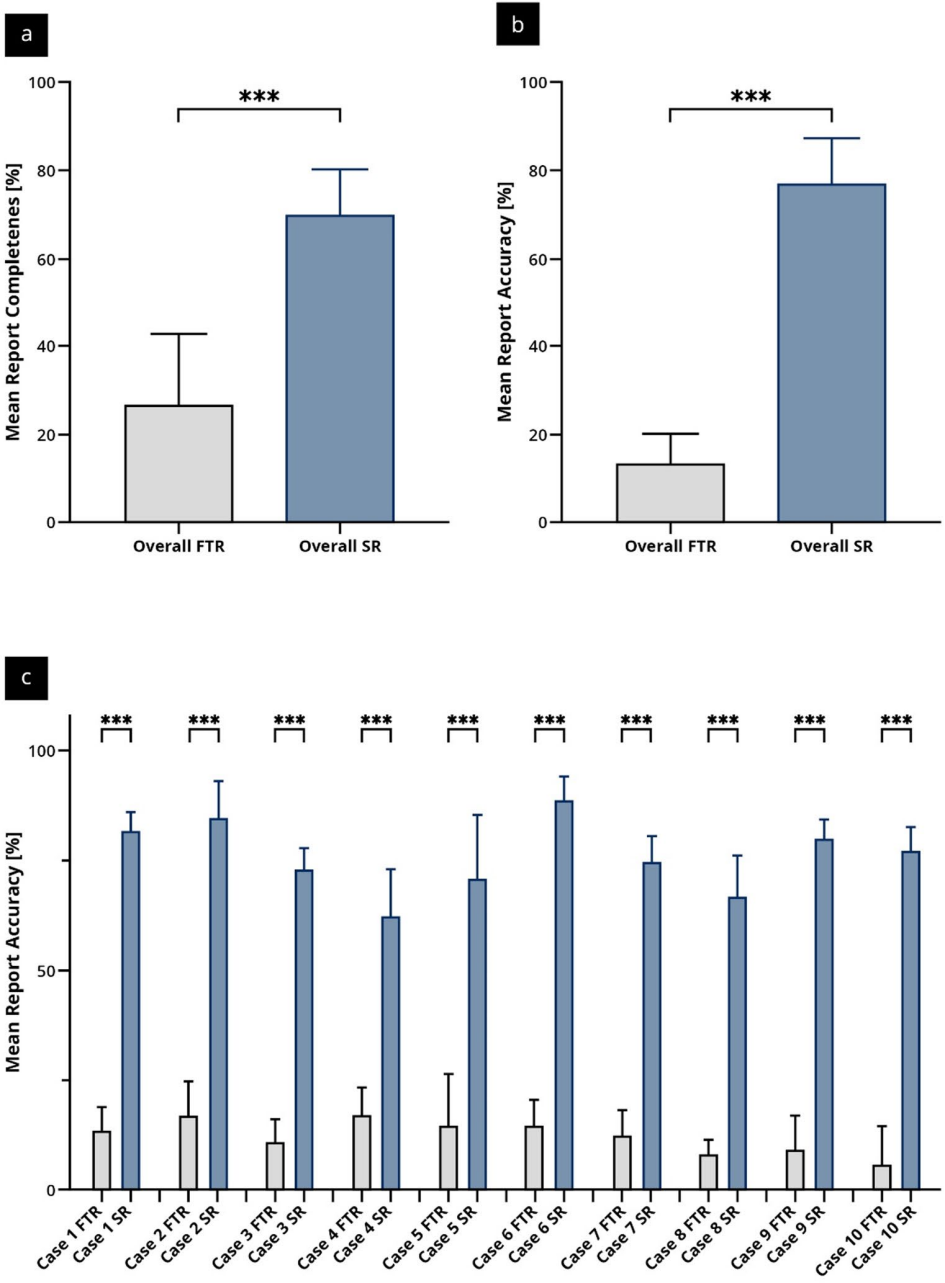
Comparison of report completeness and accuracy between structured reporting (SR) and free-text reporting (FTR); (**a**) Mean report completeness; (**b**) Mean report accuracy; (**c**) Case-by-case comparison of mean report accuracy [%] for each of the ten clinical cases. Error bars represent standard deviation. Statistical significance: *** *p* < 0.001.

Overall report completeness and accuracy as well as the case-by-case comparisons are reported as mean values ± standard deviation (Fig. 2 + Supplement 3).

SR resulted in a significantly higher overall completeness score compared to FTR (72.4 ± 13.5% vs. 20.5 ± 14.2%, *p* < 0.001). This effect was consistent across all individual cases (case 1–10), with statistically significant differences in favor of SR (*p* < 0.001 for all comparisons). For instance, completeness in Case 1 reached 89.7 ± 4.4% in the SR group compared to 32.1 ± 17.2% in the FTR group (*p* < 0.001). The smallest difference was observed in Case 10, where SR still yielded nearly double the completeness (66.5 ± 4.2% vs. 35.4 ± 12.6%, *p* < 0.001). In addition to the increased report completeness, SR markedly improved the accuracy of the reports. The overall accuracy score was significantly higher in the SR group (77.3 ± 11.6%) compared to the FTR group (12.5 ± 8.3%, *p* < 0.001). On a case-by-case basis, SR consistently produced more precise reports. For example, in case 6, accuracy reached 90.7 ± 5.4% with SR, compared to only 15.0 ± 5.7% in the FTR group (*p* < 0.001). Even in the lowest scoring SR case (case 4), the mean accuracy remained above 60% (63.5 ± 10.7%), still significantly exceeding that of the FTR group (17.5 ± 6.3%, *p* < 0.001).

### Correlations and influencing factors

**Fig. 3:**
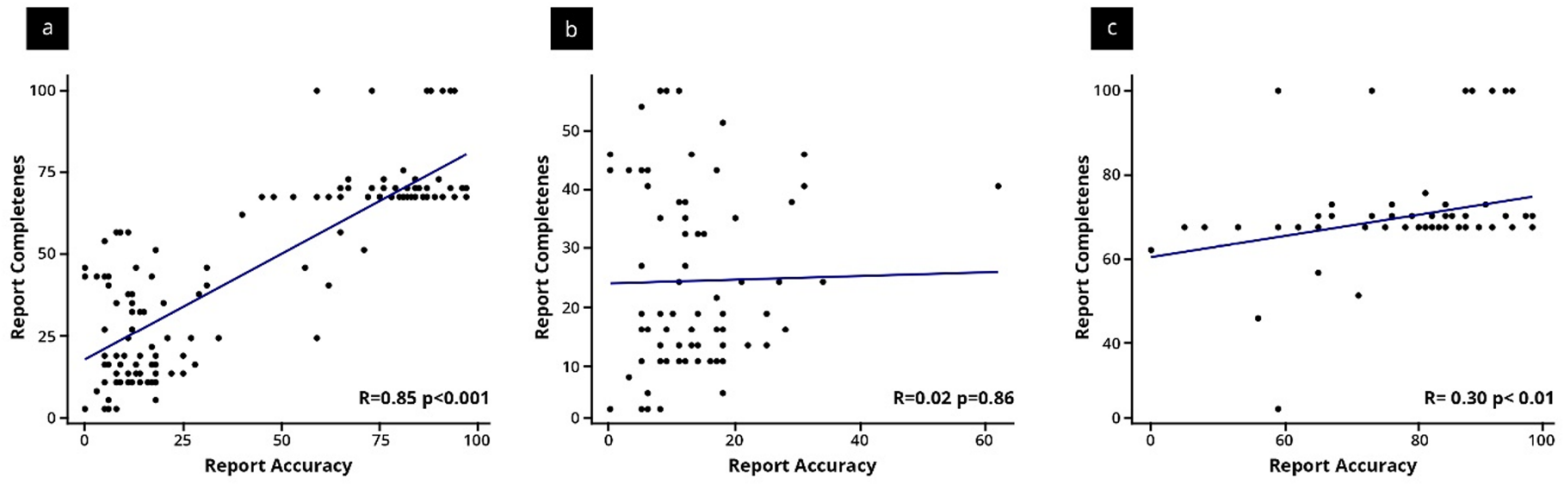
Correlation between report accuracy and completeness; (**a**) across all participants, (**b**) in the free-text reporting group (**c**) in the structured reporting group Each dot represents an individual report; the blue lines indicate linear regression trends.

A strong positive correlation (see Fig. 3) was observed between report completeness and accuracy across both reporting groups (*r* = 0.85, *p* < 0.001). However, when stratified by report type, a significant but weaker correlation was found in the structured reporting group (*r* = 0.30, *p* < 0.01), while no significant correlation was observed in the free-text group (*r* = 0.02, *p* = 0.86).

To further analyze the factors influencing report accuracy, a multiple linear regression model was applied. Utilizing structured reporting was the only independent and significant predictor of higher overall report accuracy (β = 62.2, *p* < 0.01) and report completeness (β = 47.4, *p* < 0.01), while DEGUM certification status, gender, medical specialty (ENT vs. maxillofacial surgery), and the number of independently performed ultrasound examinations showed no significant association.

## Discussion

The aim of the present study was to evaluate the impact of SR on the accuracy of HNS reports compared to FTR. Our data clearly indicate significant improvements of report completion, which is in line with previous publications, as well as report accuracy due to the implementation of SR^7,11–13,15,24,26^.

By standardizing report content, structure and terminology, SR guarantees that all important anatomical structures, especially dangerous variants, as well as features of the pathology at hand are addressed and reported upon reproducibly^15,26^.

Additionally, semantic sentences produced via SR incorporate a standardized terminology which adheres to current guidelines and recommendations^5^. Therefore, structured reporting ensures that reports and especially pathologic findings are significantly more objectifiable. Furthermore, the standardized format and language greatly increase comparability in terms of follow-up exams and especially for scientific use with regard to artificial intelligence, big data analyses and consequently deep learning technologies, as well as saving time in report generation^27^.

This is underlined by multiple studies that were able to show that SR improves the training process of HNS, decreases the incidence of misdiagnoses and enhances report quality and completeness^7,11,12,28,29^. Additionally, SR has been shown to provide a substantial IRR, a known weakness of sonography in general, therefore having a positive effect on comparability of reports as a basis for therapy monitoring and big data analysis^14,24,30^. These are huge factors in terms of interdisciplinary communication, surgical planning and therapeutic stratification, which heavily depend upon reproducible and precise reports^7,11,15^.

While there is plenty of evidence that the use of SR is associated with increased report completeness which may be grounds for improved patient care, the effect upon report accuracy, which basically means that the extent to which the correct diagnosis is reported remains largely unknown. Previous studies have put their focus mainly on overall report quality or IRR. To the best of our knowledge, there is no data concerning the mode of reporting on the report accuracy. Nonetheless, report accuracy, as defined as the overlap between any given report and a master report regarding content and correct pathological findings, offers a nuanced understanding of the diagnostic process and the recognition of the underlying disease. Our results contribute new evidence suggesting that SR enhances not only the breadth but also the depth resulting into a higher accuracy when compared to expert-based master reports. In spite of varying baseline expertise regarding HNS, the use of SR was consistently associated with significantly more precise and complete reports. As previously described, SR seems to be highly beneficial to inexperienced residents within the training process of HNS^7,13^.

Despite these positive results, there are limitations that must be acknowledged, Utilization of previously documented cases does not include the ultrasound examination itself, the foundation of the report. Consequently, generalization could be limited in clinical practice, where examination and reporting go hand in hand. Secondly, as participants of the SR group received a dedicated training on how to use the SR template in this study, this may introduce a bias due to learning or testing effects favoring the SR group. As previously described, the SR training was designed to teach participants how to use the template without giving them feedback whether they did so correctly to address this issue^7^. In addition, while the randomization process produced widely balanced participant baseline characteristics, a higher proportion of maxillofacial surgery residents were randomized to the SR group. In consequence, this may have introduced subtle bias, especially concerning pathologies which are typically treated by otorhinolaryngologists. It is important to clarify that report accuracy as defined in the present study does not equate to diagnostic accuracy in the strict clinical sense. Although reporting accuracy and diagnostic accuracy may be related, they represent distinct concepts. In this study, report accuracy reflects the correctness and reproducibility of reported items and terminology relative to an expert-derived master report, including both pertinent positive findings and clinically relevant negative findings. Consequently, higher accuracy scores may be achieved even when not all positive pathological findings are definitively identified, as the correct documentation of normal structures contributes to the overall score. This may bias results toward structured reporting. Nevertheless, this approach reflects routine ultrasound reporting practice, in which explicit exclusion of pathology is clinically relevant. At the same time, it implies that the observed improvements primarily indicate enhanced reporting consistency and content accuracy rather than definitive gains in diagnostic accuracy. This distinction is important for interpretation of the results and highlights the need for future studies focusing on pathology-centered diagnostic accuracy outcomes.

In consequence, future research should focus on the effect of SR application in real-world clinical settings including the ultrasound examination itself. While this has been done before, additional parameters such as IRR and accuracy as well as interdisciplinary communication and especially patient outcome need to be assessed. Future studies should also complement item-based accuracy metrics with pathology-specific analyses focusing on pertinent-positive accuracy to better disentangle report quality from diagnostic correctness. Additionally, implementation of artificial intelligence could substantially boost the impact of structured reporting. Yet, we need structured data in the context of big data analyses to provide input for artificial intelligence and deep learning solutions. AI driven automatization processes have a great potential to improve SR based report quality and especially efficiency^12^.

## Conclusion

In conclusion, this study offers strong evidence that utilization of SR significantly improves not only the report completeness more importantly the report accuracy of HNS reports. Comprehensive implementation of SR therefore has a great potential to standardize HNS documentation, improve clinical workflows, and ultimately lead to better patient outcomes.

## Supplementary Information

Below is the link to the electronic supplementary material.

Supplementary Material 1

Supplementary Material 2

Supplementary Material 3

Supplementary Material 4

## Data Availability

Data cannot be shared publicly because of institutional and national data policy restrictions imposed by the Ethics committee since the data contain potentially identifying study participants’ information. Data are available upon request from the Johannes Gutenberg University Mainz Medical Center (contact Johannes Weimer via weimer@uni-mainz.de) for researchers who meet the criteria for access to confidential data (please provide the manuscript title with your enquiry).

## References

[1] Wu, M. A comparative study of 200 head and neck FNAs performed by a cytopathologist with versus without ultrasound guidance: evidence for improved diagnostic value with ultrasound guidance. *Diagn. Cytopathol.* **39**, 743–751. 10.1002/dc.21460 (2011). 20949470 10.1002/dc.21460

[2] Künzel, J. et al. Multiparametric ultrasound of cervical lymph node metastases in head and neck cancer for planning non-surgical therapy. *Diagnostics* 12 (2022). 10.3390/diagnostics12081842 PMC9406677 36010193

[3] Goncalves, M. et al. Interrater Reliability of Ultrasound in the Diagnosis of Sialolithiasis. *Ultraschall Med.* **40**, 481–487. 10.1055/a-0837-0712 (2019). 30731479 10.1055/a-0837-0712

[4] Cohen, O. et al. Impact of high-quality ultrasound following community ultrasound on surgical planning and active surveillance in patients with thyroid cancer. *Clin. Endocrinol. (Oxf)*. **94**, 990–997. 10.1111/cen.14415 (2021). 33448046 10.1111/cen.14415

[5] Kunzel, J. et al. [Standard reporting for head and neck ultrasound - a proposal]. *Ultraschall Med.*10.1055/a-1810-7173 (2022). 35483868 10.1055/a-1810-7173

[6] Kunzel, J. et al. [Quality in the appraisal of head and neck sonography results in university hospitals-a random sample]. *HNO* **69**, 907–912. 10.1007/s00106-020-00989-9 (2021). 33439274 10.1007/s00106-020-00989-9 PMC8545731

[7] Ernst, B. P. et al. Impact of structured reporting on developing head and neck ultrasound skills. *BMC Med. Educ.* **19**, 102. 10.1186/s12909-019-1538-6 (2019). 30971248 10.1186/s12909-019-1538-6 PMC6458758

[8] Todsen, T. et al. Head and Neck Ultrasound - EFSUMB Training Recommendations for the Practice of Medical Ultrasound in Europe. *Ultrasound Int. Open.* **8**, E29–E34. 10.1055/a-1922-6778 (2022). 36212171 10.1055/a-1922-6778 PMC9546639

[9] Weimer, J. M. et al. Ultrasound education in the digital era: face-to-face vs. webinar-teaching of head and neck ultrasound theory—a prospective multi-center study. *Front. Med.* 12–2025. 10.3389/fmed.2025.1506260 (2025). 10.3389/fmed.2025.1506260 PMC12098340 40417682

[10] Weimer, J. M. et al. Development and Integration of DOPS as Formative Tests in Head and Neck Ultrasound Education: Proof of Concept Study for Exploration of Perceptions. *Diagnostics (Basel)*. **13**10.3390/diagnostics13040661 (2023). 10.3390/diagnostics13040661 PMC9954978 36832149

[11] Ernst, B. P. et al. Structured reporting of head and neck ultrasound examinations. *BMC Med. Imaging*. **19**, 25. 10.1186/s12880-019-0325-5 (2019). 30917796 10.1186/s12880-019-0325-5 PMC6437950

[12] Ernst, B. P. et al. The use of structured reporting of head and neck ultrasound ensures time-efficiency and report quality during residency. *Eur. Arch. Otorhinolaryngol.* **277**, 269–276. 10.1007/s00405-019-05679-z (2020). 31612337 10.1007/s00405-019-05679-z

[13] Ernst, B. P. et al. Evaluation of optimal education level to implement structured reporting into ultrasound training. *Med. Ultrason.*10.11152/mu-2530 (2020). 32905561 10.11152/mu-2530

[14] Norenberg, D. et al. Structured Reporting of Rectal Magnetic Resonance Imaging in Suspected Primary Rectal Cancer: Potential Benefits for Surgical Planning and Interdisciplinary Communication. *Invest. Radiol.* **52**, 232–239. 10.1097/RLI.0000000000000336 (2017). 27861230 10.1097/RLI.0000000000000336

[15] Ernst, B. P. et al. The role of structured reporting and structured operation planning in functional endoscopic sinus surgery. *PLoS One*. **15**, e0242804. 10.1371/journal.pone.0242804 (2020). 33253265 10.1371/journal.pone.0242804 PMC7703956

[16] Becker, S. et al. ENT Residents Benefit from a Structured Operation Planning Approach in the Training of Functional Endoscopic Sinus Surgery. *Med. (Kaunas)*. **57**10.3390/medicina57101062 (2021). 10.3390/medicina57101062 PMC8541081 34684099

[17] Lasrich, M. et al. [Increased report completeness and satisfaction with structured neurotological reporting in the interdisciplinary assessment of vertigo]. *Hno*10.1007/s00106-024-01464-5 (2024). 38592481 10.1007/s00106-024-01464-5 PMC11422286

[18] Ernst, B. et al. Structured Reporting of Head and Neck Sonography Achieves Substantial Interrater Reliability. *Ultrasound Int. Open.* **09**, E26–E32. 10.1055/a-2173-3966 (2023). 10.1055/a-2173-3966 PMC10556873 37808417

[19] Elsholtz, F. H. J. et al. Inter- and Intrareader Agreement of NI-RADS in the Interpretation of Surveillance Contrast-Enhanced CT after Treatment of Oral Cavity and Oropharyngeal Squamous Cell Carcinoma. *AJNR Am. J. Neuroradiol.* **41**, 859–865. 10.3174/ajnr.A6529 (2020). 32327436 10.3174/ajnr.A6529 PMC7228176

[20] Boulanger, S. M. et al. Investigating the reliability and validity of subacromial space measurements using ultrasound and MRI. *J. Orthop. Surg. Res.* **18**, 986. 10.1186/s13018-023-04482-1 (2023). 38135882 10.1186/s13018-023-04482-1 PMC10740303

[21] Dardenne, G. et al. Accuracy and Precision of an Ultrasound-Based Device to Measure the Pelvic Tilt in Several Positions. *J. Ultrasound Med.* **39**, 667–674. 10.1002/jum.15141 (2020). 31665548 10.1002/jum.15141

[22] Safina, A. et al. Precision imaging-its impact on image quality and diagnostic confidence in breast ultrasound examinations. *Br. J. Radiol.* **88**, 20140340. 10.1259/bjr.20140340 (2015). 26286642 10.1259/bjr.20140340 PMC4730958

[23] Menditto, A., Patriarca, M. & Magnusson, B. Understanding the meaning of accuracy, trueness and precision. *Accred. Qual. Assur.* **12**, 45–47. 10.1007/s00769-006-0191-z (2007).

[24] Ernst, B. P. et al. Structured Reporting of Head and Neck Sonography Achieves Substantial Interrater Reliability. *Ultrasound Int. Open.* **9**, E26–E32. 10.1055/a-2173-3966 (2023). 37808417 10.1055/a-2173-3966 PMC10556873

[25] Sahni, V. A. et al. Impact of a Structured Report Template on the Quality of MRI Reports for Rectal Cancer Staging. *AJR Am. J. Roentgenol.* **205**, 584–588. 10.2214/AJR.14.14053 (2015). 26295645 10.2214/AJR.14.14053

[26] Becker, S. et al. ENT Residents Benefit from a Structured Operation Planning Approach in the Training of Functional Endoscopic Sinus Surgery. *Medicina* **57**, 1062 (2021). 34684099 10.3390/medicina57101062 PMC8541081

[27] Shen, Y. T., Yue, W. W., Xu, H. X. & Editorial Ultrasound in Oncology: Application of Big Data and Artificial Intelligence. *Front. Oncol.* **11**, 819487. 10.3389/fonc.2021.819487 (2021). 35004335 10.3389/fonc.2021.819487 PMC8730332

[28] Park, S. B. et al. Structured Reporting versus Free-Text Reporting for Appendiceal Computed Tomography in Adolescents and Young Adults: Preference Survey of 594 Referring Physicians, Surgeons, and Radiologists from 20 Hospitals. *Korean J. Radiol.* **20**, 246–255. 10.3348/kjr.2018.0109 (2019). 30672164 10.3348/kjr.2018.0109 PMC6342761

[29] Tuncyurek, O. et al. Structured versus narrative reporting of pelvic MRI in perianal fistulizing disease: impact on clarity, completeness, and surgical planning. *Abdom. Radiol. (NY)*. 10.1007/s00261-018-1858-8 (2018). 10.1007/s00261-018-1858-8 30519819

[30] European Society of R. ESR paper on structured reporting in radiology. *Insights Imaging*. **9**, 1–7. 10.1007/s13244-017-0588-8 (2018). 10.1007/s13244-017-0588-8 PMC5825315 29460129

