# Correction: The influence of structured reporting on the accuracy of head and neck sonographies

**DOI:** 10.1038/s41598-026-45703-x

**Published:** 2026-03-31

**Authors:** Johannes Matthias Weimer, Julian Künzel, Christoph Raczeck, Mohamed Hodeib, Tim Koppen, Liv Weimer, Anna Levi, Maximilian Rink, Sven Becker, Benjamin Philipp Ernst

**Affiliations:** 1 Rudolf-Frey Teaching Department, University Medical Center of the Johannes Gutenberg University Mainz, Langenbeckstraße 1, 55131 Mainz, Germany; 2 Department of Internal Medicine I, University Medical Center of the Johannes Gutenberg University Mainz, Langenbeckstraße 1, 55131 Mainz, Germany; 3 Department of Otorhinolaryngology, University Hospital Regensburg, Franz-Josef-Strauß-Allee 11, 93053 Regensburg, Germany; 4 Department of Otorhinolaryngology, University Medical Center Bonn, Venusberg-Campus 1, 53127 Bonn, Germany; 5 Department of General, Visceral and Thoracic Surgery, German Armed Forces Central Hospital of Koblenz, Rübenacher Straße 170, 56072 Koblenz, Germany; 6 Department of Radiation Oncology and Radiotherapy, University Medical Center of the Johannes Gutenberg University Mainz, Langenbeckstraße 1, 55131 Mainz, Germany; 7 Department of Otorhinolaryngology, Goethe-University Frankfurt, University Medical Center, Theodor-Stern-Kai 7, 60590 Frankfurt, Germany; 8 Department of Otorhinolaryngology, Head and Neck Surgery, University of Tübingen Medical Center, Elfriede- Aulhorn-Straße 5, 72076 Tübingen, Germany

Correction to: *Scientific Reports* 10.1038/s41598-026-43561-1, published online 10 March 2026

The original version of this Article contained an error in the name of the author Christoph Raczeck, which was incorrectly given as Christoph Raczek.

The original Article has been corrected.

