# Supplementary material for: The influence of structured reporting on the accuracy of head and neck sonographies
Source: Sci Rep. 2026 Mar 10;16:8560. doi: 10.1038/s41598-026-43561-1 (PMC12976335; doi:10.1038/s41598-026-43561-1)
Supplement: Supplementary file 1 — Supplementary Material 1 [file 41598_2026_43561_MOESM1_ESM.docx]

**Supplement 1** Cases and description

| **Case** | **Description** |
| --- | --- |
| 1  Acute cervical lymphadenitis | 24-year-old female patient with a history of extraction of right wisdom tooth 4 days ago. No history of exposure to noxious agents. Increased pain for 2 days, foetor ex ore, subfebrile temperatures. According to the treating oral surgeon, there is a local wound infection without suspected abscess. Oral antibiotics have already been started with amoxicillin/clavulanic acid. The patient has now consulted an ENT specialist due to additional swelling in the right-sided angle of the jaw. |
| 2  Branchial cleft cyst | 21-year-old patient with sudden onset of painful swelling cervical left side. The pain had already eased following oral antibiotic therapy with cefuroxime prescribed by the general practitioner, but the swelling was still present. No history of exposure to noxious agents. |
| 3  Non-Hodgkin lymphoma of the neck | 31-year-old patient with progressive, indolent left cervical swelling since 3 weeks. In addition, the patient had experienced unintentional weight loss of 8 kg in the last 3 months. On request, the patient reported that he had to change his shirt 1-2 times every night due to increased sweating. No history of exposure to noxious agents. |
| 4  Tongue base tumor | 48-year-old female patient with progressive swallowing difficulties over the past 6 weeks. Two treatments with oral antibiotics had not brought any improvement. In the meantime, she is eating mainly mashed food. No history of exposure to noxious agents. In the past, gynecological treatment had been necessary due to HPV-16-associated mucosal changes. |
| 5  Sialolithiasis with duct obstruction | 55-year-old patient with postprandial pain and swelling of the left parotid region that had been present for 2 days. The swelling would decrease approximately one hour after eating. No history of exposure to noxious agents. |
| 6  Pleomorphic adenoma of the parotid gland | 61-year-old patient with a known, slowly progressive right parotid mass, which he first noticed about a year ago while shaving. No history of exposure to noxious agents. |
| 7  Plunging ranula | 22-year-old patient with swelling in the anterior floor of the mouth as well as submentally that appeared 2 weeks ago. No infections remembered. No previous illnesses. No history of exposure to noxious agents. |
| 8  Multifragmentary sialolithiasis | 65-year-old female patient with postprandial pain and a swelling in the left submandibular and parotid region since yesterday. The swelling would decrease about one hour after eating. No history of exposure to noxious agents. |
| 9  Peritonsillar abscess | 20-year-old female patient with acute tonsillitis that has been present for 5 days and is being treated with oral penicillin. Since today massive pain exacerbation only on the left side with reduced jaw opening and uvula edema. |
| 10  Hashimoto’s thyroiditis | 26-year-old female patient presenting for thyroid assessment complaining about fatigue and weight gain for several months. Laboratory tests revealed hypothyroidism with positive TPO antibodies. |
