# Supplementary material for: The influence of structured reporting on the accuracy of head and neck sonographies
Source: Sci Rep. 2026 Mar 10;16:8560. doi: 10.1038/s41598-026-43561-1 (PMC12976335; doi:10.1038/s41598-026-43561-1)
Supplement: Supplementary file 2 — Supplementary Material 2 [file 41598_2026_43561_MOESM2_ESM.docx]

**Supplement 2 Sample evaluation case 4**

**Document: SR-Report**

**Participant code:**

**Case: 4**

| **Participant report** | **Master report** |
| --- | --- |
| **report**  Lymph nodes: The neck vascular nerve sheath on both sides showed no evidence of pathologic lymph nodes or other masses.  submandibular gland The submandibular gland appeared normal on both sides without evidence of space-occupying lesions, stones or congestion.  Base of the tongue: In the base of the tongue, on the right side at the junction with the tonsillar lodge, there was a diffuse lesion with a size of 2.87 mm x 2.25 mm, hypoechoic, inhomogeneous, blurred border. Overall unclear dignity.  Tonsillar loge: The above-mentioned mass was found in the tonsillar loge | **report**  Lymph nodes: The neck vascular nerve sheath showed no evidence of pathologic lymph nodes or other masses on both sides.  Major cervical vessels: The major cervical vessels were unobstructed and showed a regular flow pattern.  Parotid gland: The parotid gland appeared normal on both sides with no evidence of space occupying lesions, stones or congestion.  Submandibular gland: The submandibular gland appeared normal on both sides without evidence of space occupying lesions, stones or congestion.  Sublingual gland: The sublingual gland appeared normal on both sides without evidence of space occupying lesions, stones or congestion.  Thyroid gland: The thyroid glandules appeared regular on both sides without evidence of goiter or space occupying lesions.  Base of tongue: A solitary, inhomogeneous, ill-defined mass with a size of 28.7 mm x 22.5 mm was found on the right side of the base of the tongue, suspiciously malignant.  Tonsillar loge: The tonsillar loge was symmetrical on both sides without evidence of a space occupying lesion or an abscess. |

**Completeness score case 4**

|  | Description | Points possible | Points achieved |
| --- | --- | --- | --- |
| Lymph node levels | Level Ia-VI were described and anomalies were ruled out | 12 | 12 |
| Major cervical vessels | described and anomalies were ruled out | 8 | 0 |
| Parotid gland | described on both sides | 2 | 0 |
| Submandibular gland | described on both sides | 2 | 2 |
| Sublingual gland | described on both sides | 2 | 0 |
| Thyroid gland | described on both sides | 2 | 0 |
| Base of tongue | described on both sides | 2 | 2 |
| Tonsillar loge | described on both sides | 2 | 2 |
| readability |  | 5 | 5 |
| overall |  | 37 | 23 |

**Accuracy score – Case 4**

|  |  |  |  |  |  |  | Points possible | Points achieved |
| --- | --- | --- | --- | --- | --- | --- | --- | --- |
| Category**→**  Items **↓** | Neck vascular nerve sheat | Both sides | Space occupying lesions | Pathology |  |  |  |  |
| Lymph nodes | mentioned | mentioned | none | none |  |  | 4 | 4 |
| Category**→**  Items **↓** | **Flow pattern** | **Obstruction** |  |  |  |  |  |  |
| Major cervical vessels | Regular | none |  |  |  |  | 2 | 0 |
| Category**→**  Items **↓** | **appearance** | **Space occupying lesions** | **Stone** | **congestion** |  |  |  |  |
| Parotid gland | regular on both sides | none | none | none |  |  | 4 | 0 |
| Submandibular gland | regular on both sides t | none | none | none |  |  | 4 | 4 |
| Sublingual gland | regular on both sides | none | none | none |  |  | 4 | 0 |
| Category**→**  Items **↓** | **appearance** | **Space occupying lesions** | **goiter** |  |  |  |  |  |
| thyroid gland | regular on both sides | none | none |  |  |  | 3 | 0 |
| Category**→**  Items **↓** | **Number of pathologic findings** | **size** | **Localization** | **Border definition** | **Echogenity** | **Pathology** |  |  |
| base of tongue | One, No | 28,7 x 22,5mm  yes | Right side  yes | ill-defined  yes | Inhomogeneous  yes | Malignant  yes | 6 | 5 |
| Category**→**  Items **↓** | **Appearance** | **Space occupying lesions** | **Abscess** |  |  |  |  |  |
| Tonsillar lodge | regular on both sides | none | none |  |  |  | 3 | 0 |
| **overall** |  |  |  |  |  |  | 30 | 13 |
