# Supplementary material for: The influence of structured reporting on the accuracy of head and neck sonographies
Source: Sci Rep. 2026 Mar 10;16:8560. doi: 10.1038/s41598-026-43561-1 (PMC12976335; doi:10.1038/s41598-026-43561-1)
Supplement: Supplementary file 3 — Supplementary Material 3 [file 41598_2026_43561_MOESM3_ESM.docx]

Supplement 3 Results on Completeness and Accuracy

| **Characteristics** | **FTR** | **SR** | **p - value** |
| --- | --- | --- | --- |
| **Completeness score** (n, mean ± SD in %) | n = 116 20.5 ± 14.2 | n = 140 72.4 ± 13.5 | <0.0001 |
| Case 1 | n = 8, 32.1 ± 17.2 | n = 11, 89.7 ± 4.4 | <0.0001 |
| Case 2 | n = 11, 19.3 ±15.1 | n = 14, 74.1 ± 14.3 | <0.0001 |
| Case 3 | n = 12, 24.8 ± 17.3 | n = 16, 73.4 ± 8.5 | <0.0001 |
| Case 4 | n = 10, 25.4 ± 13.6 | n = 17, 68.0 ± 11.4 | <0.0001 |
| Case 5 | n = 18, 17.0 ± 12.2 | n = 18, 64.5 ± 11.6 | <0.0001 |
| Case 6 | n = 10, 20.8 ± 17.6 | n = 15, 67.6 ± 0.2 | <0.0001 |
| Case 7 | n = 11, 23.4 ± 10.5 | n = 7, 73.0 ± 2.2 | <0.0001 |
| Case 8 | n = 10, 23.0 ± 7.6 | n = 9, 68.5 ± 1.4 | <0.0001 |
| Case 9 | n = 13, 25.4 ± 19.8 | n = 15, 67.6 ± 0.2 | <0.0001 |
| Case 10 | n = 13 35.4 ± 12.6 | n = 18, 66.5 ± 4.2 | <0.0001 |
| **Precision score** (n, mean ± SD in %) | n = 115, 12.5 ± 8.3 | n = 141, 77.3 ± 11.6 |  |
| Case 1 | n = 8, 13.6 ± 5.6 | n = 11, 83.4 ± 4,2 | <0.0001 |
| Case 2 | n = 11, 17.1 ± 7.5 | n = 14, 76.5 ± 8.4 | <0.0001 |
| Case 3 | n = 12, 11.2 ± 5.2 | n = 16, 74.4± 5.0 | <0.0001 |
| Case 4 | n = 10, 17.5 ± 6.3 | n = 17, 63.5 ± 10.7 | <0.0001 |
| Case 5 | n = 18, 15.1 ± 11.7 | n = 18, 72.6 ± 14.3 | <0.0001 |
| Case 6 | n = 10, 15.0 ± 5.7 | n = 15, 90.7 ± 5.4 | <0.0001 |
| Case 7 | n = 11, 13.1 ± 5.4 | n = 7, 76.1 ± 5.6 | <0.0001 |
| Case 8 | n = 10, 8.5 ± 2.7 | n = 9, 68.1 ± 9.1 | <0.0001 |
| Case 9 | n = 13, 9.4 ± 7.6 | n = 15, 81.6 ± 4.4 | <0.0001 |
| Case 10 | n = 13, 6.0 ± 8.6 | n = 18, 79.1 ± 5.1 | <0.0001 |
