# Supplementary material for: The influence of structured reporting on the accuracy of head and neck sonographies
Source: Sci Rep. 2026 Mar 10;16:8560. doi: 10.1038/s41598-026-43561-1 (PMC12976335; doi:10.1038/s41598-026-43561-1)
Supplement: Supplementary file 4 — Supplementary Material 4 [file 41598_2026_43561_MOESM4_ESM.pdf]

Study Protocol

Title
The Influence of Structured Reporting on the Accuracy and Completeness of Head and Neck Sonography Reports: A Prospective Randomized Educational Study

Protocol Version and Date
- Version: 1.0
- Date: January 2023
- Status: Final (prepared prior to study initiation)

Trial Registration
This study was conducted as a prospective randomized educational study without patient-level interventions.
In accordance with German and international regulations, trial registration was not mandatory because:
- No patients were recruited or randomized
- No diagnostic or therapeutic interventions were performed
- Only anonymized, pre-existing ultrasound cases were used
- Participants were physicians in postgraduate training

Therefore, no clinical trial registry entry exists.
This information can be stated verbatim in the cover letter and manuscript.

Sponsor
None (investigator-initiated study).

Funding
This study received no external funding.

Ethics Approval
Ethical approval was obtained prior to study initiation:
- Ethics Committee: Ethik-Kommission der Landesärztekammer Rheinland-Pfalz
- Reference Number: 2018-13225
- Approval Date: prior to January 2023

All procedures complied with the Declaration of Helsinki and national regulations.

Study Objectives
Primary Objective
To evaluate whether structured reporting (SR) improves:
1. Report accuracy
2. Report completeness

in head and neck sonography compared to free-text reporting (FTR).
Secondary Objectives
- To analyze the relationship between report completeness and report accuracy
- To identify independent predictors of report accuracy and completeness
- To assess whether examiner experience influences reporting performance

Klinik für Hals-Nasen-Ohrenheilkunde
http://www.unimedizin-ffm.de
Direktor:
Prof. Dr. T. Stöver
Sekretariat: J. Droese
Tel.: 069 / 6301-5163/6788
Fax: 069 / 6301-5435
Mail: zhno.sekretariat@unimedizin-ffm.de

Stellvertretender Direktor:
PD Dr. med. M. Leinung

Bearbeiter: J. Droese
Datum: 28. February 2026

Privat-Sprechstunde
Prof. Dr. med. T. Stöver
Tel. 069 / 6301-5163

Allgemeine Poliklinik
Tel. 069 / 6301-3313
Mo. - Fr. 08.00 bis 12.00 Uhr

Notfallambulanz
Tel. 069 / 6301-5362

Spezialsprechstunden
Tel. 069 / 6301-3313

- **Mittelohr und Trachea**
  Mo. 08.00 bis 15.00 Uhr
- **Nasen- und Nasennebenhöhlen**
  Di. 08.00 bis 15.00 Uhr
- **Allgemeine HNO und Allergologie**
  Mi. 08.00 bis 15.00 Uhr
- **Onkologische Sprechstunde**
  Do. 08.00 bis 15.00 Uhr
- **Plastische Chirurgie**
  Fr. 08.00 bis 12.00 Uhr

Hörzentrum
Tel. 069 / 6301-3726

- **Hörimplantat-Sprechstunde**
  Mo. - Do.
  08.00 bis 15.00 Uhr

Audiologische Akustik
Prof. Dr.-Ing. U. Baumann
Terminvereinbarung:
Tel. 069 / 6301-6896

Phoniatrie & Pädaudiologie
Tel. 069 / 6301-5775

Study Design

- Design: Prospective, randomized, controlled educational study
- Setting: DEGUM-certified head and neck ultrasound courses
- Study Period: 2023–2024
- Allocation Ratio: 1:1 (FTR vs. SR)
- Blinding:
  - Participants: not blinded (reporting format known)
  - Evaluators: fully blinded to reporting format and participant identity

Participants
Eligibility Criteria
Inclusion Criteria

- Participation in a DEGUM-certified head and neck ultrasound course
- Written informed consent
- Completion of at least one assigned reporting case

Exclusion Criteria

- Missing informed consent
- Incomplete report submission

Randomization and Allocation
Participants were randomly assigned to one of two groups:

- Free-Text Reporting (FTR) Group
- Structured Reporting (SR) Group

Randomization was performed using a computer-generated random allocation sequence prior to case assignment.
Baseline characteristics (age, gender, specialty, ultrasound experience, DEGUM level) were recorded before randomization.

Study Materials
Clinical Cases

- Number of cases: 10
- Content: Typical head and neck pathologies
- Materials provided:
  - Clinical history
  - Representative ultrasound images
- Patient population: Adult patients (≥18 years)

Cases were created by board-certified otorhinolaryngologists with DEGUM Level III certification and based on real clinical cases.

Interventions
Free-Text Reporting (FTR)
Participants created reports using a conventional free-text format routinely used in ultrasound courses.
Structured Reporting (SR)
Participants used a standardized, web-based structured reporting template developed according to current DEGUM recommendations.
Before reporting, SR participants received a standardized technical introduction to the reporting software without feedback on report correctness.

Outcomes
Primary Endpoints

1. Report Completeness
   - Definition: Proportion of required anatomical regions and report elements explicitly mentioned
   - Scoring: Item-based, independent of correctness
2. Report Accuracy
   - Definition: Correctness of reported findings compared to expert-derived master reports
   - Includes:
     - Correct identification of pathology
     - Correct exclusion of pathology
     - Accurate terminology and characterization

Reference Standard (Master Reports)
For each case, an expert-derived master report was created by DEGUM Level III-certified otorhinolaryngologists and served as the reference standard.
Master reports included:

- All relevant pathological findings
- Explicit documentation of normal anatomical structures

Report Evaluation

- All reports were anonymized
- Evaluated independently by two blinded DEGUM-certified experts
- Case-specific evaluation templates were derived from the master reports
- Discrete, predefined items were scored
- Weighted scoring reflected diagnostic relevance

Scores were normalized to percentage values for comparability.

Sample Size Calculation
Sample size calculation was performed a priori based on expected differences in report accuracy:

- Expected accuracy ≥80%:
  - FTR: 50%
  - SR: 70%
- Power: 80%
- Alpha: 0.05

Required minimum sample size: 186 reports (93 per group)

Statistical Analysis Plan

- Descriptive statistics for baseline characteristics
- Normality testing: Shapiro–Wilk test
- Group comparisons:
  - Unpaired t-test (parametric)
  - Chi-squared test (categorical variables)
- Correlation analysis:
  - Pearson correlation between completeness and accuracy
- Multivariate linear regression:
  - Dependent variables: completeness, accuracy
  - Independent variables: reporting format, experience, specialty, gender, DEGUM level
- Significance level: $p < 0.05$

Statistical analyses were conducted using GraphPad Prism 10.

Data Management and Confidentiality

- All data were anonymized before analysis
- Digital storage complied with institutional data protection policies
- Data access restricted to study investigators

Data Availability

Due to ethics committee restrictions, data cannot be shared publicly. Data are available upon reasonable request from the corresponding institution.

Dissemination Plan

Results were intended for publication in a peer-reviewed scientific journal and presentation at academic conferences.
